# Selective Ultrasonic Gravimetric Sensors Based on Capacitive Micromachined Ultrasound Transducer Structure—A Review

**DOI:** 10.3390/s20123554

**Published:** 2020-06-23

**Authors:** Dovydas Barauskas, Mindaugas Dzikaras, Dovydas Bieliauskas, Donatas Pelenis, Gailius Vanagas, Darius Viržonis

**Affiliations:** Kaunas University of Technology, Panevėžys Institute of Technologies and Business, LT-37164 Panevėžys, Lithuania; mindaugas.dzikaras@ktu.edu (M.D.); Dovydas.Bieliauskas@ktu.edu (D.B.); Donatas.Pelenis@ktu.edu (D.P.); Gailius.Vanagas@ktu.edu (G.V.)

**Keywords:** Capacitive Micromachined Ultrasound Transducer, gas sensing, molecule sensing, Micro Electro Mechanical System, microfabrication, finite element modeling

## Abstract

This review paper discusses the advances of the gravimetric detection devices based on capacitive micromachined ultrasound transducers structure. Principles of gravimetric operation and device modeling are reviewed through the presentation of an analytical, one-dimensional model and finite element modeling. Additionally, the most common fabrication techniques, including sacrificial release and wafer bonding, are discussed for advantages for gravimetric sensing. As functional materials are the most important part of the selective gravimetric sensing, the review of different functional material properties and coating and application methods is necessary. Particularly, absorption and desorption mechanisms of functional materials, like methylated polyethyleneimine, with examples of applications for gas sensing and using immune complexes for specific biomolecules detection are reviewed.

## 1. Introduction

Application of ultrasonic transducers for sensing has become popular among multiple areas that include sensing of various physical, chemical, and biochemical phenomena. While several microelectromechanical system (MEMS) structures, like cantilevers, bridges, thin-film, or printed piezoelectric devices, can be applied for the ultrasonic sensing, the capacitive micromachined ultrasound transducers (CMUT) concept is highly advantageous due to the unprecedented sensitivity potential, good integration with electronics, hermetic active structure, high redundancy of the resonators, and CMOS (Complementary Metal-Oxide-Semiconductor) compatible fabrication process [1,2,3]. Gravimetric detection is a method that determines the quantity of the analyte (the molecule, species, or material of interest) by determining its mass. The most widely known gravimetric sensor is a Quartz Crystal Microbalance (QCM), where the resonating structure of bulk piezoelectric material is excited at a resonance frequency, which is subject to change when some molecules, vapors, or other species are deposited on the working surface of the device. For selective detection of the analyte, the resonator surface is usually precoated with the functional material, which specifically binds the target molecules and is indifferent to the rest of ambient materials or molecules. This establishes the basic concept of the selective ultrasonic gravimetric sensor: functional film plus electromechanical detection structure. 

Because there are two parts of a selective ultrasonic gravimetric sensor (SUGS), the properties of each of them have an impact on the sensor’s performance. Usually, there are several characteristic properties that describe the performance: sensitivity, the limit of detection, and cross-selectivity. Here, sensitivity describes the steepness of the main sensor function, usually characterized as a mass unit per the frequency unit. For this parameter, CMUT structure has little competition, since it was demonstrated by several groups to reach numbers down to 10^−18^ g/Hz and below [2,3,4,5,6,7,8]. As for the other two parameters, they are more related to the properties of the functional material of the sensor [1,7,9,10,11,12], and the topic of the functional materials suitable for SUGS remains highly underexplored.

Gas sensing is the most natural field of application of SUGS with a CMUT structure due to the extremely low moving mass of the electromechanical part of devices. At the same time, recently, gas sensing has earned increased attention in academia, industry, and households due to its environmental importance and increased security demand [13,14,15,16,17,18,19,20,21]. It became a subject of interest and significance spreading over many different areas, such as industrial production—methane detection in mines or the detection of volatile organic compounds (VOCs) [22], automotive industry—detection of dangerous gas emissions from vehicles, medical applications and healthcare—study of the environment [23] (greenhouse effect gases [24,25,26]), air quality monitoring [27], analysis of breath, or food degradation and spoilage [28,29].

While gas sensor development is mainly driven by environment monitoring, primarily the gases that have the highest global warming potential (GWP), a measure that tells how much of the heat is trapped in the atmosphere relative to carbon dioxide, are within the research interest [30]. The gases with the highest GWP are listed in Table 1, describing their GWP as well as the lifetime in the atmosphere [31,32,33]. CO_2_ is constantly present in the Earth’s atmosphere and is a major participant in the greenhouse effect, shown to be the culprit of global warming [34]. Although other gases are at smaller concentrations, however, compared to CO_2_, they have a much higher GWP and their lifetimes in the atmosphere are several hundred times larger than CO_2_’s, making their contribution to the global warming effect even more substantial.

The detection and monitoring of these gases are complicated, and researchers have been developing complex systems in order to create fast, precise, simple, portable, and low-cost sensing platforms [35,36]. Some of the developed systems try to miniaturize a technology that is extensively used for quantifying various gas mixtures—gas chromatography—while maintaining similar or even better efficiency when compared to laboratory-based tests [36]. However, reaching such goals has been proven to be difficult since micro detectors with an acceptable lower limit of detection for such systems are still in development [37]. Some researchers have switched their focus to microelectromechanical systems (MEMS) for creating reliable, sensitive, and highly selective gas sensing platforms. Many research and market reviewers recognize the absence of the MEMS-based class of gas sensors on the market [28]. It is important to recognize that most commercially available sensors are based on electrochemical sensing principles, which detection is based on the measuring of the charge generation or conductivity changes. MEMS elements, such as suspended micro membranes, microbridges, and cantilevers, improved some characteristics of chemical transistors and resistors [38], such as power dissipation and the operational temperature ranges. When these EMEMS structures are being used as electrochemical sensors in combination with metals, metal oxides, or nanomaterials, response times can be also improved [23,39,40]. Another issue with the electrochemical type of sensors is their limited selectivity since multiple different gases have similar electrochemical interactions with the sensor’s active sensing area [41]. The sensitivity and selectivity problems of electrochemical sensors are just partly solved by the MEMS technologies, like lowering the sensor operational temperature by 50% or reducing the response time to tens of seconds [23]. 

Recently, it was demonstrated [42] that by introducing SUGS with CMUT structure and using the proper sensing materials it is possible to overcome many drawbacks of the other technologies in the device properties such as sensitivity, cross-selectivity, and limit of detection. Gas sensors that employ the gravimetric detection techniques (i.e., sensors of the mass absorbed onto the resonating structure) were reviewed recently [37]; however, despite the wide scope of the review, less attention was paid for the limit of detection and cross-selectivity characteristics of the sensors. SUGS research features also the development of a functional material layer, which binds or absorbs gas molecules. Therefore, this particular class of sensors can bring the art of gas and molecular sensing to the unprecedented detection limits and cross-selectivity.

## 2. Electromechanical Part

### 2.1. CMUT Structure and Working Principle

**Figure 1:**
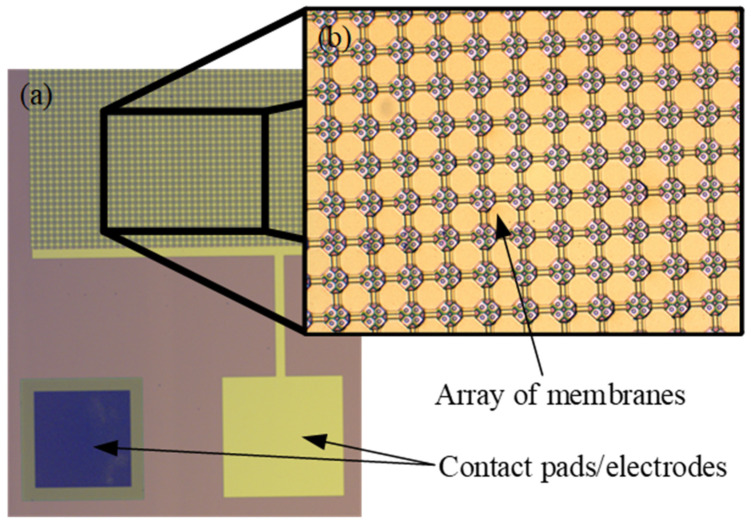
(**a**) Micrograph of capacitive micromachined ultrasound transducer (CMUT) array of membranes with top electrodes and contact pads; (**b**) A close-up micrograph of CMUT membranes fabricated using surface micromachining sacrificial release method.

Capacitive micromachined ultrasound transducers are typically manufactured using micromachining techniques specifically for silicon MEMS. The device structure has an array of membranes, usually called the capacitive cells, and these membranes are vacuum-backed (a vacuum gap in between the top and bottom electrodes), connected in parallel, and can be easily actuated with electrostatic forces by applying alternating current (AC) to the top and bottom electrodes. A complete CMUT structure and the close-up image is shown in Figure 1. The small size of the membranes allows high frequency resonance at the range of tens of MHz together with the quality factors of several hundred, meaning specifically designed structures for specific applications make excellent, high-resolution mass sensing platforms [43,44,45,46,47]. Moreover, signal-to-noise ratio can be easily improved by simply implementing these structures in large arrays [48], which would reduce the possibility of some false positive detection events.

### 2.2. Theoretical Background for the CMUT-Based SUGS

**Figure 2:**
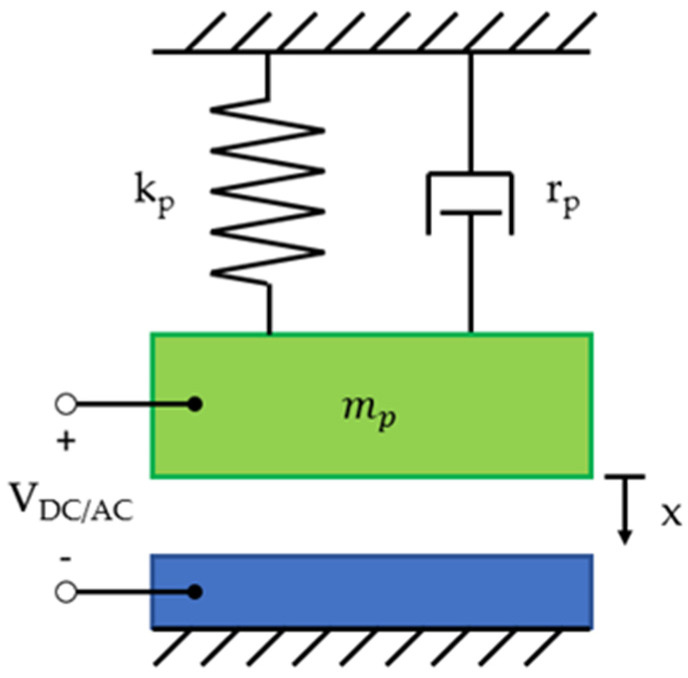
Mass-spring-damper model of a CMUT [49].

Different modeling methods of CMUTs are chosen according to the requirements of solution accuracy, computational resources, and an appropriate variety of data postprocessing capabilities. The modeling of CMUTs is challenging due to multiple fields of physics involved in its operation, namely mechanics, acoustics, structural mechanics, electrostatics, and electrodynamics [49]. However, the theoretical background of the CMUT operation can be easily explained by modeling a simplified one-dimensional case of a parallel plate capacitor. Here the mechanics are approximated by a mass-spring-damper system. The simplified mass-spring-damper model of CMUT is shown in Figure 2.

The top electrode is attracted to the bottom electrode under direct current (DC) bias, oscillations can be excited by the alternating current (AC), whereas at equilibrium, the mechanical spring force of the membrane counters the electrostatic force. Most conventional CMUT devices are biased by comparatively large DC voltage and modulated by a small AC voltage. If the top electrode is moved by a distance x, the capacitance of the parallel plate capacitor can be given by:

(1) $$C\left(x\right)=\frac{A\varepsilon_{0}\varepsilon_{r}}{g_{eff}-x}$$

(2) $$g_{eff}=\frac{t_{i}+t_{m}}{\varepsilon_{r}+g_{0}}$$

where A is the top electrode area, $\varepsilon_{0}$ is the vacuum permittivity, $\varepsilon_{r}$ is the insulator relative permittivity, $g_{eff}$ is the effective gap height, $g_{0}$ is the initial gap under zero bias voltage, $t_{i}$ is the thickness of the insulator layer, and $t_{m}$ is the thickness of the membrane [1]. The dynamics of the model can be solved via Newton’s law of motion:

(3) $$m_{p}\frac{d^{2}x}{dt^{2}}+r_{p}\frac{dx}{dt}+k_{p}x=f_{el}+f_{ac}-f_{p_{0}}$$

where $m_{p}$ is the mass constant, $r_{p}$ is the damping constant, $k_{p}$ is the spring constant, $f_{el}$ is the electric loading force, $f_{ac}$ is the acoustic loading force, and $f_{p_{0}}$ is the force due to atmospheric pressure $p_{0}$ [1].

This model is ideal in situations where a quick solution is needed for general characteristics of CMUT sensor such as collapse voltage, membrane displacement, and electrodynamics [50,51]. Furthermore, the principles of parallel plate capacitor theory are utilized in finite element modeling of CMUTs, where it is segmented into many parallel plate capacitors, which allows an approximation of the electrostatic force that is applied to each element [49,52,53,54]. On the other hand, the analytical model does not provide an analysis of complex acoustic interactions and all the underlying physics. Therefore, the model in the present form is used only in the early stages or very specific cases of CMUT development and research [49,55].

**Figure 3:**
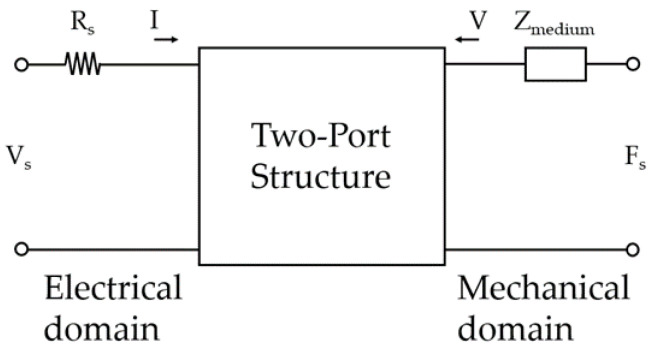
Generalized two-port network model [1].

More sophisticated models are using the two-port network, with the analytical parallel plate capacitor model inside. This two-port network can be viewed as an electromechanical transducer with the electrical energy supplied to the electric port and the complex mechanical load connected to the mechanical port, as shown in Figure 3.

Here, $V_{S}$ is the input voltage source,$\;R_{S}$ is the electrical resistance of input voltage source, $F_{S}=pA$ is the source of force due to acoustic pressure, I is current, V is the velocity, and $Z_{medium}$ is the impedance of the medium in which the transducer operates and it can be considered as $Z_{medium}=Z_{a}A$ ($Z_{a}$ is the characteristic acoustic impedance of the plate) [49].

This type of analysis is valid under small signal conditions where the membrane displacement does not reach the collapse regime and insignificant spring softening is caused by the bias voltage. It can be used to calculate resonance frequency, electromechanical impedance, and the small signal receives and transmits sensitivities as a function of frequency [49,55,56]. Furthermore, the maximum signal output pressure can also be calculated using the resonant frequency of the membrane. Most importantly for SUGS development, the complex impedance in this model can be used to represent the loading of the CMUT structure by the analyte with full selection of the physical properties: specific gravity, viscosity, and elastic modulus. Although this method presents a significantly wider range of CMUT characteristics that can be obtained compared to the unidimensional parallel plate capacitor, it falls short in providing the solution over the non-linearities of CMUT structure [49].

In cases where the most sophisticated analysis of CMUT devices is needed, the finite element analysis (FEA) method is used. The FEA modeling in all cases follows a similar procedure that begins with the geometry definition, then subdividing it into smaller elements (finite elements), over which the governing physical equations are used to find the solution. FEA allows modeling of CMUT structure in two- and three-dimensional space with including different physical domains in a single digital experiment, for example, electrostatics, acoustics, and fluidics [57]. Static, modal, harmonic, or time-domain analysis can be performed depending on what performance capabilities have to be tested [49,52,53]. Furthermore, finite element modeling allows the analysis of CMUTs under collapsed conditions where stress stiffening effects due to large membrane displacement are present [53,54,58]. Using finite element analysis, entire CMUT arrays can also be modeled through the application of a wave-guide model where a single cell is created but with periodic boundary conditions [49,53,58,59]. Due to the versatility of the FEA method, there are very few limitations that could be attributed to it. The most significant issue, related to the finite element method is related to the computational resources available, which creates a limit on how complicated and accurate solutions can be obtained. Additionally, simulations cannot replace experiments entirely, because they usually fail to include unexpected changes in the fabrication precision, material properties, and other real-world conditions [49,53,59].

### 2.3. CMUT Fabrication Methods

During the last decades, several different microfabrication methods have been developed for manufacturing capacitive micromachined ultrasound transducers [60,61]. One of the methods includes basic surface micromachining techniques like photolithography, dry and wet etching, chemical vapor deposition, and lift-off processes. Photolithography can be used to pattern the desired design of the device and electrical connections [9,62,63,64]. Dry and wet etching is used to create cavities, open contact pads for electrical connections, or separate devices with trenches [65,66,67,68,69,70]. Material deposition using evaporators [71,72] or chemical vapor deposition techniques such as LPCVD (Low Pressure Chemical Vapor Deposition) [73,74] or PECVD (Plasma Enhanced Chemical Vapor Deposition) [71,72,73,75] are used to form either protective layers on top of metalized electrodes or produce critical structures, like CMUT membranes itself. Some of the more complicated fabrication techniques involve specific processing steps such as chemical–mechanical polishing [76,77], deep reactive ion etching [78], local oxidation (LOCOS) process [79,80], and Oxford cryogenic etching of silicon [81].

#### 2.3.1. Sacrificial Release CMUT Fabrication Method

**Figure 4:**
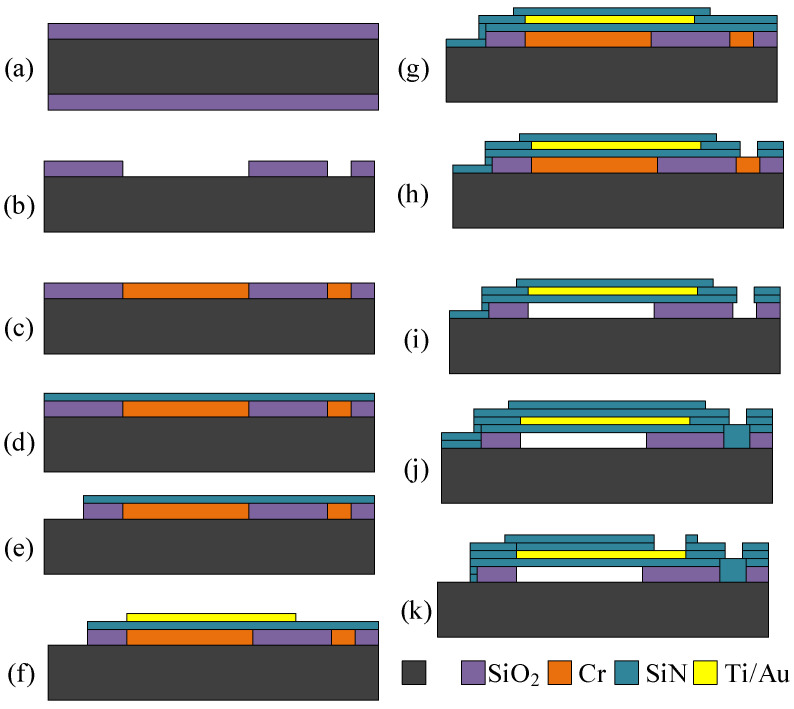
Sacrificial release CMUT device fabrication steps: (**a**) thermal oxidation, (**b**) cavity formation, (**c**) sacrificial layer deposition (Cr), (**d**) membrane formation using silicon nitride PECVD deposition, (**e**) ground electrode openings, (**f**) top electrode formation, lift-off manufacturing step, (**g**) passivation with PECVD SiN, (**h**) etch hole formation, (**i**) etching of the sacrificial layer, (**j**) closing etch holes with PECVD SiN, and (**k**) revealing top and bottom electrodes.

The very first method that was developed for CMUT fabrication was the sacrificial release [82]. It has a specifically selected material that acts as the sacrificial one during the processing steps. The gap where the membrane can freely move in this case is formed by an etching step that creates a cavity between the top and the bottom plate and later etching holes are vacuum-sealed to allow the devices to be used in liquids. An example illustration of sacrificial release processing steps is given in Figure 4. Silicon wafer is oxidized with a silicon dioxide layer (Figure 4a) and this layer is photolithographically patterned and wet etched to create cavities (Figure 4b). The sacrificial layer is shown in Figure 4c [83]. Then a silicon nitride layer is added using the PECVD process to form the membrane layer (Figure 4d). To decrease the residual stress for the membrane, a mix of low frequency and high frequency of PECVD Si_x_N_y_ can be used [84]. Figure 4e shows a lithography step where the bottom electrode is revealed by etching through silicon nitride and the oxide layers using dry etch equipment. Vacuum evaporation step is performed to establish the top electrode on top of the membranes as shown in Figure 4f. Another SiN layer is deposited to protect the top electrode during sacrificial release (Figure 4g). Later, a dry etching step using RIE (Reactive Ion Etch) is performed to create holes for the etchant to go into and etch away the sacrificial layer (Figure 4h). After the sacrificial release process (Figure 4i), one of two processes can be applied to successfully release the free-standing membranes: critical point drying or freeze drying. Both methods dry the devices without exerting any residual stress on the small, fragile, and sensitive microstructures. Then, the etch holes are covered with another SiN layer (Figure 4j) sealing the device and making it possible to be used in immersion applications. Finally, the contact pads for bottom and the top electrode are opened (Figure 4k). By using this method, the vacuum gap is determined by the sacrificial layer thickness and the top moving membrane is defined by the thickness of the combined thickness of Si_x_N_y_ and the thickness of the top electrode. The main advantage of this method is comparatively low cost and relatively low processing temperatures, which allow for post-CMOS integration. The main disadvantage is comparatively low uniformity of the deposition and etching processes and limited quality of multiple lithography steps [49]. 

#### 2.3.2. The Basic Wafer Bonding Method

**Figure 5:**
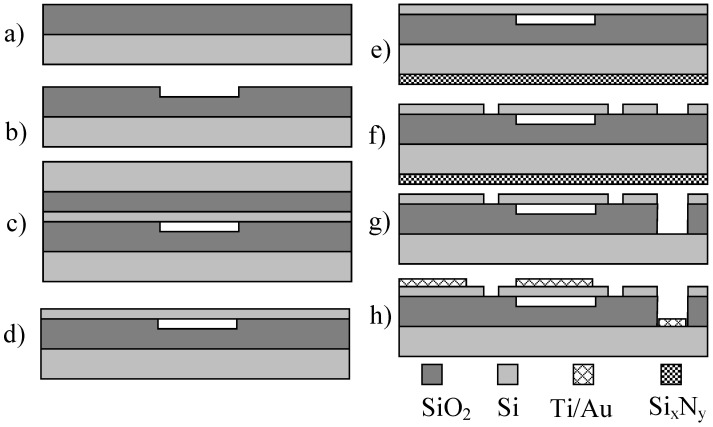
Wafer bonding CMUT device fabrication steps: (**a**) thermal oxidation, (**b**) cavity formation, (**c**) wafer bonding, (**d**) handle and buried oxide removal, (**e**) backside protection layer, (**f**) device and membrane separation, DRIE (Deep Reactive Ion Etch) or Oxford, (**g**) contact formation, and (**h**) metallization, electrode formation.

The most basic and more commonly used fabrication method for CMUTs is wafer bonding [80,85]. Using this technique requires combined technologies of surface micromachining and silicon-on-insulator (SOI) technology. Wafer bonding method adds advantages that include the uniformity and control of plate thickness and surface roughness. An example of such microfabrication technology steps is shown in Figure 5. A heavily-doped Si wafer is covered by SiO_2_ by thermal oxidation (Figure 5a), and UV photolithography is performed to etch cavities that determine the device vacuum gap height (Figure 5b). The second oxidation step passivates the exposed Si and can prevent short circuits when the membrane is working in collapse mode. Then, direct bonding with subsequent annealing of both wafers in high temperature (1100 ^˚^C) (Figure 5c) is done. To release the CMUT membranes, the bonded SOI wafer carrier is ground down with chemical mechanical polishing (CMP) step and finished with wet etching steps (Figure 5d). Before further processing, a backside protection layer, usually PECVD SiN, is deposited (Figure 5e). Devices then can be separated by using a deep silicon etching commonly known as Bosch [78] or Oxford process (Figure 5f). When devices are separated, their contact pads are revealed using another photolithography step and an etch step (Figure 5g). Metal connections for top or bottom electrodes are formed photolithographically with evaporation and lift-off procedures (Figure 5h). As the last step, an isolation layer can be used to cover the membranes and passivate the sidewalls, making them possible to be used in immersion applications [1,63,86]. 

When compared to the sacrificial release method, wafer bonding fabrication technique has its advantages in the uniformity and control over the membrane thickness and gap height, offering high reproducibility with almost no residual stress acting on the membrane. However, it is more costly due to manufacturing steps requiring expensive silicon-on-insulator wafers, and some of the direct bonding methods used are difficult and very sensitive to multiple parameters, such as cleanliness and surface roughness. Suitable SOI wafers are difficult to manufacture so their cost of producing one is high when compared to similar wafers needed for sacrificial release method. Researchers have shown that silicon nitride can be used to cover a standard wafer, and it can be bonded to substrate wafer [87]. Additionally, some research groups have already shown promising and interesting alternative CMUT fabrication methods [60,88,89].

#### 2.3.3. Advanced Wafer Bonding Methods

Direct silicon nitride bonding with a single local oxidation of silicon (LOCOS) process is a wafer bonding technique offering even better vacuum gap height optimization and can reduce the parasitic capacitance compared to the standard wafer bonding process. With this process, it has been shown that low voltage devices with a gap height of 40 nm can be fabricated [80]. The thickness uniformity achieved by this process can lead to higher quality factors, which is an important property of SUGS employing CMUT structure.

Modified wafer bonding techniques have also been used by multiple researchers that specifically target CMUT performance aspects like charging [90], which can reduce the reliability of devices [84,91,92,93]. Higher reliability, simplified processing, and better surface roughness tolerances can be achieved by anodic bonding and using glass to bond to a substrate [94,95]. More recently, the CMUT devices have started to shift toward flexibility [96] and or bendability by creating arrays from flexible components or implementing the rigid components into the flexible medium.

#### 2.3.4. Commonly Used Materials for CMUT Fabrication

The most popular and common CMUT fabrication methods described in the previous section such as wafer bonding and sacrificial release mainly rely on thin film deposition, etching, and photolithography to fabricate moving micro membranes [82]. The most common and widely used materials for CMUT and their properties are given in Table 2. The CMUT membranes are usually made out of silicon, SiC, Si_3_Ni_4_, and polysilicon, and the top electrodes are typically made out of aluminum or other highly conductive materials. Structural materials are chosen due to their excellent electrical and mechanical properties, allowing the minimization of the effective gap between the bottom and top electrodes, ensuring electrical stability and maximizing the elasticity modulus. By doing so, the electric field concentration can be maximized and the impedance matching with the electronics can be improved. Although recently some researchers have successfully fabricated and tested polymer-based membranes [97]. 

## 3. Functional Materials

Fabrication of CMUT structure is only a halfway of producing the functional SUGS device because the functional layer is needed to selectively absorb target molecules from air or another medium. The most promising class of functional materials are polymers [98]. Polymers used to functionalize SUGS can be conducting [99] or nonconducting [100]. The essential requirement for the functional layer is high selectivity and sensitivity to the molecules of interest. For biosensing applications, one part of the immune complex can be used to functionalize SUGS and make it specifically sensitive to another part of a complex [1,62,63,86,101]. One can use commercially available or custom designed functional materials for device surface functionalization [4,22].

The viability of SUGS based on the CMUT structure was proven several times since previous decades. Very high resolution or limit of detection was demonstrated for DMMP [5], organic compounds [3], and CO_2_ [9,10] detection. Additionally, the CMUT-based SUGS concept was proven to be suitable for biosensing [11,101]. However, there is only a limited selection of materials and CMUT structure functionalized methods researched. It is commonly understood that mass-loading of the resonant sensing structure, the resonant frequency will shift [94,95,96,97], and in most cases, developers tend to prove the linear relationship between the shift of the resonant frequency to the quantity of molecules of interest. This is understandable since SUGS operation can be maintained then by tracing the frequency of the free-running oscillator [102]. However, there can be several different interactions between the gas molecules and the functional layer. For example, a chemical reaction between the gas molecules and the functional layer can express itself by changing the properties of the layer differently, as it was shown while researching the interaction between methylated polyethyleneimine (mPEI) functionalized CMUT and SO_2_ gas molecules [42]. It was also proven there that because of different interactions the same functional layer has high cross-selectivity potential when CO_2_ and SO_2_ gases are to be distinguished. The capability of CMUT-based SUGS to react to the complex changes of the functional layer is a unique property, which empowers even greater research interests for the functional material of SUGS.

### 3.1. Surface Functionalization Methods

The modification of the device surface for gravimetric sensing can be done in many different ways [99]. The most common methods of thin films (presumably, polymer films) deposition on the surface of the SUGS include electrochemical deposition, dip-coating, spin-coating, Langmuir–Blodgett technique, layer-by-layer self-assembly technique, thermal evaporation, vapor deposition polymerization, drop-coating, and inkjet printing.

Thin-film formation with layer-by-layer (LbL) technology process is a promising deposition technique since it is based on the deposition of alternatively charged materials that are laid down with wash steps in between and does not require any electrical currents. This is done using simple techniques such as immersion, spinning, spraying, electromagnetism, or even fluidics [103,104,105,106,107,108,109].

Another method for creating thin polymer films is thermal evaporation and vapor deposition polymerization. This method requires high temperatures to successfully create a thin layer of polymer on top of the device’s structure. This technique is not as suitable as other techniques for CMOS integrated CMUTs, because using of additional thermal processes can damage the integrated electronics part of a device [110,111].

The simplest functionalization method is a drop-coating technique that uses a handheld pipetting device. The main disadvantages: polymer solution droplet formation with spontaneous solvent evaporation requires precision, controlling the thickness and acceptable uniformity levels are difficult, and the method is limited to comparatively large area coverage. The main advantage is that this method is amazingly simple with almost no waste of materials. Because this method does not have reproducibility of layer deposition in terms of thickness or sensing area coverage [6], it would not be the best surface functionalization technique.

**Figure 6:**
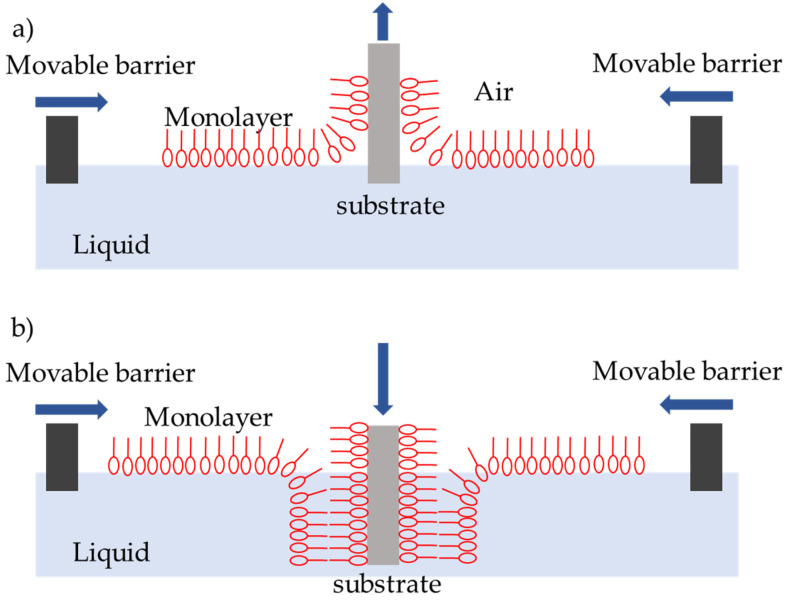
Langmuir–Blodgett thin film deposition: (**a**) first layer deposition on a hydrophilic surface; (**b**) second layer deposition on a hydrophobic layer.

Another more complex thin polymer layer deposition technique is called by the name of Langmuir–Blodgett film [112,113,114,115,116] named after Irving Langmuir and Katharine B. Blodgett. This film can contain one or more thin monolayers of organic materials that are deposited using a simple immersion (or emersion after being submerged) technique allowing deposition from the surface of a liquid onto a solid body or already-deposited substrate Langmuir–Blodgett film as shown in Figure 6. The technique behind it is that a monolayer is adsorbed with each immersion (emersion) step homogenously. This means that the controllability of layer thickness is extremely high and very accurate thin layers can be formed. The accuracy comes from the knowledge of each monolayer’s layer thickness. These monolayers are usually assembled vertically with respect to the surface, composed of amphiphilic molecules meaning a molecule that has a hydrophilic head and hydrophobic tail, e.g., fatty acids. Creating thin controllable polymer layers using the Langmuir–Blodgett deposition technique would allow one to increase the reproducibility of the active layer on the device giving properties that are needed for gas sensing applications. The main disadvantage of such thin layer formation is that specific amphiphilic or other self-assembling molecules are required, usually, no covalent bonding to the surface is done so the formed monolayer degrades rather quickly.

Electrochemical deposition requires electric current and electrically conductive surface in order to reduce metal cations, so they could adhere to an electrode and form a thin coating. Usually, electrochemical functionalization of MEMS systems with polymers [117,118,119] is used to modify the surface of microcantilevers using a standard three-electrode configuration [120]. It is an advantage of CMUT structure, which is insensitive to the electrical currents produced during electrochemical deposition, which may damage more fragile types of MEMS.

Another functionalization method is a simple spin coating [121,122,123] of the device using a spinner. Using this method, the reproducibility in terms of layer thickness and coverage is very high, but it suffers from the inability to cover multiple areas with different polymers on the same sensing device, decreasing the sensing selectivity and reducing the possible multichannel performance of such resonating devices. 

Thus, we come to the last method for functionalizing the CMUT device’s active zone surface layer of a microdevice—inkjet dispensing. Inkjet dispensing method allows the user to obtain more uniform layers and functionalize specific parts of the device with precision and control of the dispensed volume. When droplets from the inkjet printer dry out, they leave a uniform thin polymer on the surface of the device. Compared to the spin coating method, the inkjet-dispensing method does not have such high uniformity, but it can selectively pattern different device sensing areas creating a single die array with multiple devices covered with different polymers [124].

The most preferable surface modification method is either spin coating or inkjet dispensing due to their high reproducibility of the surface layer parameters such as thickness. Highly available apparatus such as spin coaters and inkjet printers, including 3.5D printers (3D printers with extreme resolution and special extruder, for example, a micro syringe for dispensing micro droplets of the polymer film) is widely available for researchers and is the main driving topic for further research interests.

### 3.2. Materials for Surface Functionalization and Gas Sensing Applications

For the early and fast detection of different gases in the atmosphere, various sensors have been developed that integrate polymers [125]. Sometimes these polymers are accompanied by zeolites that are used as commercial adsorbents or catalysts due to their porous structure, which creates an extremely high surface area and is highly advantageous as gas adsorption medium [4,126]. 

Some of the more commonly used materials for gravimetric gas sensing are polymers such as polyvinyl alcohol (PVZ), polyethylene oxide (PEO), polyethyleneimine (PEI), polyepichlorodyhrin, polycaprolactone (PECH), phenylmethyldiphenylsilicone (PCL), poly-4-vinylphenol (OV-25), and polyvinylpyrrolidone (PVP) [127]. These polymers, in combination with CMUTs, have already been shown to be able to detect various gases such as methanol, ethanol, isopropanol, and acetone vapor [128], successfully proving that the CMUT technology-based gas sensing systems can be developed. Moreover, other researchers have shown that gas sensing can be done using other types of devices (microcantilevers, nanopillars, and nanotubes) with polymers, such as polyaniline, polythiopene, polyfuran, polypyrole, and polyphenylenevinylene [99,126,129]. A Khuri-Yakub et al. have shown that detection of volatile organic compounds (more specifically, acetone, toluene, isoprene, and styrene) can be done with a silicon micro-ring device modified with a polyesteramid and poly(2-vinyl pyridine), with detection as low as 100 parts per billion (ppb) [130]. 

Nanocomposites are also a great candidate for being a specific modification material for increasing the selectivity of a gravimetric gas detection system. Researchers have shown that acetone detection can be significantly improved by fabricating polymeric nanocomposites consisting of amine-terminated silicon nanoparticles (Si NPs-NH_2_) and poly (4-vinylphenol) on a micro-gap interdigitated electrode, and the response of the sensor can be significantly improved by the addition of silicon nanoparticles to the polymer layer [131].

It was also shown that by using different functional layers such as PVA, PECH (Polycaprolactone), PCL (Phenylmethyldiphenylsilicone), PVP (Polyvinylpyrrolidone), PEI (Polyethyleneimine) and functionalizing different parts of the sensing area or different arrays specifically designed for that layer, a selective gas detection system can be created [9,12,128,132,133,134]. Some designs of multichannel sensing systems described in previous research articles include multiple arrays, reference devices, trenches, and channels for negating noise coming from the environmental factor or crosstalk between the neighboring arrays.

Some polymers are extremely important due to their possible use cases in the detection of environmental pollutants sulfur, carbon, and nitrogen oxides. For example, methylated polyethyleneimine (mPEI) have been used for surface functionalized CMUT devices in order to detect CO_2_ and SO_2_ gas molecules [7]. Furthermore, the surface recovery of the functional layers has not been fully researched and multiple questions such as how many cycles can the absorption surface sustain before it loses its properties need to be answered. Although, some researchers have already shown that the functional layer can withstand multiple tests for more than a hundred times of absorption desorption cycles [4,8].

Analysis of the literature regarding the polymers that are suitable for gas sensing led to the conclusion that there is an abundant type of different materials that are useful or can be used in gas sensing or biosensing applications. The different polymers and synthesis methods of materials available that are not fully understood are still being researched.

### 3.3. Absorption Mechanisms of Functional Materials

**Figure 7:**
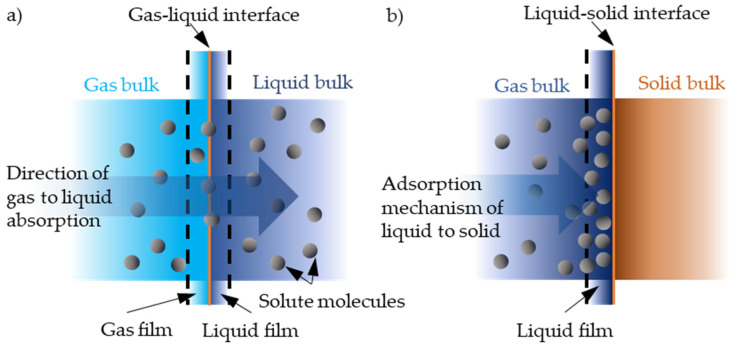
Gas–liquid absorption (**a**) and liquid–solid adsorption (**b**) mechanism [75]. Grey spheres represent solute molecules absorbing or adsorbing from one interface to the other.

The term absorption is defined as the diffusion of molecules throughout the bulk of the solid or liquid. Adsorption is defined as the accumulation of the molecular species at the surface of the phase rather than in the bulk of the solid or liquid. The main difference is either the process takes place on the surface or inside the bulk of the material. This means that absorption is a bulk phenomenon and adsorption is a surface phenomenon and therefore, has different kinetics. Both processes are regarded as sorption processes as seen in Figure 7 [135].

The main adsorption mechanism at the solid–gas interface, the main concepts, and thermodynamics are closely detailed in the reference [136], and Fourier transform infrared spectroscopy (FT-IR) can be used to investigate these gas absorption mechanisms [7,137,138].

For the functionalized gas sensing devices, the absorption happens when the gas of interest diffuses into the polymer film on the device changing its mass, which simultaneously changes the resonance frequency. Most of the time, when the device is purged with nonspecific gases, the bound analyte gas molecules diffuse out of the polymer film, restoring the polymer mass and recovering the original resonant frequency [4]; however, this interaction is not observed all of the time and is highly dependent on the functional material and physical and chemical interactions between them. The chemical interaction taking place between the analyte species and the functional layer is extremely important because the relationship between the gas concentration and the resonance frequency shift highly depends on that interaction. Furthermore, it allows us to determine the possibilities of the restoration of the unabsorbed polymer and further device use. Depending on how fast the gas diffuses, diffusion distance, which is based on the molecule size, size of analyte cavities, etc., the membrane mass, when the membrane vibrates, changes based on these interactions.

More in-depth research regarding the molecular interactions of absorption layer and the gases are being done in order to determine the mechanisms of which the gravimetric sensing technologies are responsible for sensing [7].

### 3.4. Gas and Biomolecule Sensing with SUGS Featuring CMUT Structure

**Figure 8:**
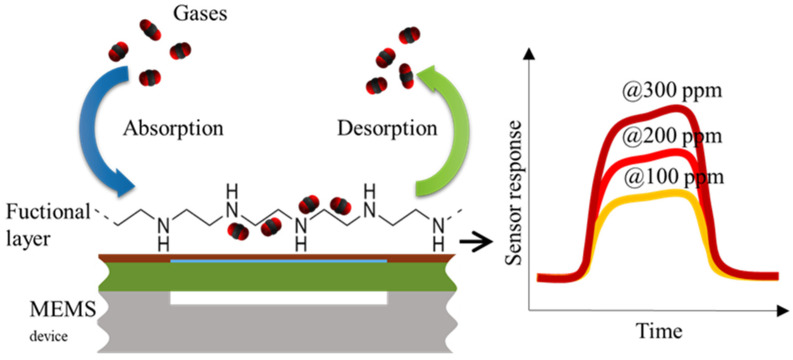
Main working principle of a gravimetric gas sensor using a functional layer and microelectromechanical system (MEMS) device. The figure shows how the gas molecules are absorbed and desorbed over time when there is a gradient of gases of interest and the main sensor response over time with various concentration values.

The main working principle of a CMUT device for gravimetric gas sensing application is shown in Figure 8. The sensor’s response highly depends on the gas molecule interaction with the specifically selected functional layer and how it reacts to the increase and decrease in various gas concentrations either through absorption, desorption, or chemical interactions [7,42]. The functional layer saturates with the reactive gas molecules and the change in device parameters such as resonance frequency stabilizes according to the concentration values. The typical dynamic ranges of concentrations and limits of detection are highly variable and are highly dependent on the functional layer that is used for the mass absorption effect.

Biosensing with SUGS has the advantage of avoiding molecule-labeling steps because of the change of the state of the functional layer during specific interaction with the analyte (for example, while there is an interaction of antigen and antibody) will provide even more complex electromechanical signal. There are specific challenges of SUGS to be applied for biodetection, mostly related to the overdamping of the resonator by the liquids. Additionally, in biosensing, biomolecules often are suspended in a liquid. This is typically avoided by allowing the biochemistry to take place in a liquid phase and drying out the transducer, which regenerates the optimal characteristics for performing the measurements [1,11].

Lee et al. have demonstrated a CMUT-based biosensor for the detection of neuropeptide somatostatin (SST), which is related to the development of Alzheimer’s and other neurodegenerative diseases and is found in patients in low concentrations [139]. Their approach involved covering a gold-coated CMUT surface with a monolayer of cysteine-modified protein G and attaching the SST-binding IgG antibodies on top. The immobilized SST-specific antibodies can then capture the SST target molecules from the sample solution. The most pronounced frequency shift for this device was with an SST concentration of 10 pg/ml – 1 ng/ml, which is six orders of magnitude lower concentration than for any previously reported SST biosensors.

Eaimkhong et al. have shown that CMUTs can be used for the detection of another biomarker, TNF-α, a tumor necrosis factor present as a marker in cardiovascular diseases [11]. Their constructed sensor has a clinically relevant linear response in the range of 10–1000 ng/µl of TNF-α. This range corresponds to 10–100 kHz shift in signal from the CMUT resonant frequency of 50–52 MHz. TNF-α was attached to the chip surface using biotin-streptavidin chemistry. The CMUT chip silicon surface was first exposed to the biotinylated bovine serum albumin (BSA), then to streptavidin, and subsequently to anti-TNF-α (a TNF-α antibody). Even though the sensor does not exceed the sensitivity of conventional antibody assays, such as ELISA, the direct, label-free, cost, small amounts of testing material, detection time, and the potential to improve the sensitivity by scaling up the CMUT array are beneficial. The group has also demonstrated that the developed system can work as an immunological assay by firstly binding an antihuman IgG antigen and exposing it to the human IgG antibody.

Our group has also demonstrated CMUT use for immunological label-free detection of antibodies binding to surface-functionalized bovine leukemia virus protein gp51 [62,101]. The detection was done with whole blood samples and CMUTs with a resonant frequency of 12 MHz. This research has also demonstrated that two parameters—namely the resonance frequency shift and the shift in electrochemical impedance—can be observed for better interrogation of biomolecular binding processes. The sensor detection speed also outperformed electrochemical sensors.

The gravimetric CMUT-based biosensors have the potential to replace labeled assays; however, the drying step requires additional processing time, is incompatible with most of the microfluidic designs, and limits the device reuse due to surface-functionalized protein denaturation. Recently, Pelenis et al. have demonstrated a nongravimetric CMUT-based biosensor working in liquid phase [64,140]. The sensor uses CMUTs for generating and receiving acoustic signals through Scholte type waves above a gold surface, functionalized with bovine serum albumin (BSA), precipitated from the liquid. As the waves propagate through the liquid medium, they become retarded due to the interaction with surface bound BSA. That results in decreased wave time of flight and lowered signal. The researchers have shown that BSA can be detected down to 0.1 mg/ml concentration when exposed to 10 mm by 10 mm detection area. The sensor is potentially conducive for microfluidic integration, multiple analyte detection, and most importantly retaining original liquid medium throughout the process; however, wave retardation-based sensors require specific geometries and are still in the early research phase.

Electrochemical sensors are dominating the research in label-free biosensor development due to their costs and simpler electronics. However, SUGS, and specifically, CMUT-based SUGS have distinct advantages over electrochemical sensors, such as much lower limit of detection, detection speed, and improved detection electronics. More advancements need to be carried out for elimination of dry steps, enabling multiple biomolecule detection, optimization of binding techniques, and many others before any commercial CMUT biosensors will be available, but the potential has been demonstrated in varied ways.

### 3.5. Measurement of the CMUT Gravimetric Sensor

The main SUGS device characteristic is its sensitivity. It highly depends on the mass of the resonating membrane and its resonance frequency. Sensitivity can be increased by having a resonating micro membrane at a high frequency with very low mass. It was already shown by other researchers that a theoretical sensitivity of 0.45 Hz/ag can be achieved by a CMUT-based gravimetric sensor device [2,3]. The sensitivity can be optimized based on the selected resonance frequency by decreasing the mass of the resonating plate, which can easily be achieved by making them as thin as possible. Some researchers have already shown that it is possible to fabricate thin plates in the range of 100 nm. Furthermore, a single CMUT-based sensor typically consists of hundreds or thousands of electrically connected cells in parallel. This lowers the overall impedance on the electronics and improves the signal to noise ratio [6]. Adding a functional layer on the CMUT gravimetric sensor allows one to selectively detect the concentration of analyte that is proportional to the additional mass and results in resonance frequency shift. Typically, coating the entire sensor area with uniform functional layer thickness results in an increase in the total mass of the movable membrane leading to decreased sensitivity due to the decrease in resonance frequency. To achieve the best results for sensitivity, a trade-off between the functional layer thickness and sensitivity requirements is necessary.

## 4. Discussion and Conclusions

Among the arguments towards CMU-based SUGS, which are presented in this review, another important factor deciding the practical value of different gravimetric detection systems is the ratio between detectable mass and active surface area. Some of the parameters regarding different types of gravimetric systems or devices are summarized in Table 2. Another MEMS-based gravimetric devices, excluding the CMUTs, are the cantilever or ring-based microstructures with possibilities of displacement for resonant characterization. NEMS-based gravimetric detection devices are nanometer sized resonant structures specifically designed for resonant characterization in nanometer scale, capable of sensing extremely small changes in mass.

Other gravimetric sensors, such as bulk acoustic wave devices (BAW) or surface acoustic wave devices (SAW), designed specifically for gravimetric detection rely on different measurement parameters, such as the propagation velocity of acoustic waves. While these kinds of devices can achieve a low level of detection limits, they are prone to many drawbacks, such as complex readout and interpretation of the signals. The comparison (see Table 3) shows that both BAW and SAW devices need the largest active surface area, which makes them quite expensive to produce. Moreover, even with the higher active surface area, mass resolution of CMUT devices can be better by several orders of magnitude when comparing to N/MEMS or other BAW, SAW-based gravimetric sensors. The availability of various CMUT designs and various fabrication methods that can be used to mass produce these devices can be seen as an advantage over the other types of gravimetric sensing devices. 

Most of the time, the resonance frequency stability of a resonating device can be determined by the Allan deviation parameter (a parameter that shows frequency stability of an oscillator over time), but when the devices are scaled down the deviation tends to increase, thus exhibiting similar and comparable performance to the most commonly used quartz crystal microbalance gravimetric detection method. CMUTs having the microarray structure with high element count (membranes or multiple arrays of membranes) can have the benefit of performance at a low concentration of target gas. The quality factor of such devices is improved by the square root of number of the elements available on the device improving its SNR ratio at the same time. The Allan deviation with the higher quality factor devices are orders of magnitude lower, allowing the devices to achieve a mass resolution in the zeptogram range.

As it was described previously, the potential of modifying device surface with specific absorbing and adsorbing materials, enables these devices to selectively detect different molecules. When considering the mass resolution and the active device area, BAW and SAW devices take the lead in performance with mass resolution and large active surface area. NEMS arrays can be advantageous over CMUTs with their better performance factors such as lower active surface area, much lower driving voltage, but in NEMS there’s a tradeoff between active surface area and effective mass resolution. Additionally, most importantly, NEMS devices are much more sensitive to various functionalization technologies, which excludes them from practical competition with CMUT-based SUGS.

Response time is also a very important parameter for gravimetric detection systems since the possible applications are mainly real-time monitoring so a quick response to the changes in gas mixture is needed [37]. See the comparison between different gravimetric detection technologies in Table 4. The longer the lag, the less data about the saturation limits are acquired. Additionally, absorption and desorption kinetics might not be the same and depend on the partial pressure (and absolute pressure) of surrounding target gases and other gases. In the case of CMUT, this can be optimized by the design of the electromechanical system such as membrane size, which would determine the absorption response, thickness would determine the frequency and the shift in frequency response, shape would increase or decrease the response due to possible absorption and desorption profiles that are based on shape, a number of membranes improve response if at least several membranes show a shift in resonant frequency, number of arrays increases active surface area improving response in this way, electrodes, contacts, material it is made of, gaps, trenches, and various reference channels can improve the response time by removing the environmental noise from the sensing system. Gas flow rate, operating temperature, and size of the environment have a strong influence on response and recovery rates. In addition, the thickness of the sensing layer, its microstructure (porosity), will also influence the response/recovery rates. Based on the sensing mechanism, response time will be highly dependent on diffusion in and out of the sensing film.

For real world applications, CMUT SUGS should retain good measuring capabilities over long periods of time. They should exhibit reversibility to avoid sensor saturation and unpredictable drift of measurement data. Material degradation is another concern. The DMMP-detecting CMUT SUGS with a proprietary polymer was shown to be fully reversible and stable for at least 250 min with 9 exposures to decreasing concentrations of DMMP [5]. Seok et al. have observed fully reversible ethanol detection for 50 min, exposing the device to nine different concentrations [133]. Other sensors have only been demonstrated for single minutes ranges [102,127]. Research on these aspects is relatively scarce and focus is typically applied to primary concerns, such as demonstration of working principles, and signal processing. Any stability issues will likely be a research topic for specific gas-sensing and layer combination on the road to commercialization.

The future direction for CMUT sensors seems to be moving towards mobile and flexible applications, making cheap, selective, multichannel, small, mobile systems [128,133,143,144,145]. There is a plethora of commercialization potential for the technologies, especially in the improvements of fabrication technologies. There is a market for smart, mobile devices for environmental sensing applications, especially smart devices for home use and monitoring. The recent development towards mobile highly flexible and integrated systems shows commercialization potential in the area of mobile applications moreover including environmental monitoring and in house easy to use measurement solutions. SUGS would fit perfectly in the category of environmental monitoring applications.

In this review, we have shown the great potential of CMUT-based SUGS, especially for gaseous species detection. Soon they are to become a key element in extremely sensitive, selective, responsive, and cost-effective sensing systems. However, the bottleneck here is the development and research of functionalization materials. Significant input from the polymer chemistry would considerably speed up the implementation of the reviewed technology. Therefore, the codevelopment of electromechanical and functional materials and functionalization methods will be the driving topic in the forthcoming years.

## Figures and Tables

**Table 1:** Gases with the highest global warming potential (GWP) [31].

Molecule	Molecule Formula	Global Warming Potential (GWP)	Lifetime in Atmosphere
**Carbon Dioxide**	CO_2_	1	Various years
**Methane**	CH_4_	25	12 years
**Nitrous Oxide**	N_2_O	298	114 years
**Fluorinated Gases**	HFCs, PFCs, SF_6_, NF_3_	HFCs: 12–14,800 PFCs: 7390–12,200 NF_3_: 17,200 SF_6_: 22,800	HFCs: 1–270 years PFCs: 2600–50,000 years NF_3_: 740 years SF_6_: 3200 years

**Table 2:** Most common CMUT membrane materials and their dielectric constants.

Membrane Material	Dielectric Constant, ε
**Silicon**	11.68
**SiC**	6.5–10
**Silicon nitride**	7–8
**Polysilicon**	29.5

**Table 3:** Gravimetric detection methods and their parameter comparison.

Technique	Active Surface	Mass Resolution
**CMUT** [5,8]	0.25–0.09 mm^2^	0.01–3×10^−3^ ng/cm^2^
**MEMS** [141]	7.3×10^−6^ mm^2^	0.1 ng/cm^2^
**NEMS** [142]	8.5×10^−8^ mm^2^	0.1 ng/cm^2^
**Other (BAW, SAW)** [37]	0.25–4 mm^2^	0.03–1 ng/cm^2^

**Table 4:** Gravimetric detection methods and their response time comparison.

Sensing Method/Material	Response Time	Source
**Electrochemical**	1–2 s	[146]
**QCM**	7–118 s	[147]
**CMUT**	20–200 s	[5]
**BAW**	<1 s	[148]
**SAW**	150–250 s	[149]
**TDLAS**	1 s	[150]
**SiC-based sensors**	0.005 s	[151]
**One dimensional nanostructured metal-oxides**	30–40 s	[152]
**Nanocrystalline f-doped SnO_2_ films**	32–65 s	[153]
**Electrocatalytic**	<15 s	
